# Smad4-Shh-Nfic Signaling Cascade–Mediated Epithelial-Mesenchymal Interaction Is Crucial in Regulating Tooth Root Development

**DOI:** 10.1359/jbmr.091103

**Published:** 2009-11-02

**Authors:** Xiaofeng Huang, Xun Xu, Pablo Bringas, Yee Ping Hung, Yang Chai

**Affiliations:** 1 Center for Craniofacial Molecular Biology, School of Dentistry, University of Southern California Los Angeles, CA, USA; 2 Department of Stomatology, Beijing Friendship Hospital, Capital Medical University Beijing, China

**Keywords:** *SMAD4*, tooth root development, TGF-β/BMP, hers, shh, *Nfic*, mouse

## Abstract

Transforming growth factor β (TGF-β)/bone morphogenetic protein (BMP) signaling is crucial for regulating epithelial-mesenchymal interaction during organogenesis, and the canonical Smad pathway–mediated TGF-β/BMP signaling plays important roles during development and disease. During tooth development, dental epithelial cells, known as *Hertwig*'*s epithelial root sheath* (HERS), participate in root formation following crown development. However, the functional significance of HERS in regulating root development remains unknown. In this study we investigated the signaling mechanism of Smad4, the common Smad for TGF-β/BMP signaling, in HERS in regulating root development. Tissue-specific inactivation of *Smad4* in HERS results in abnormal enamel and dentin formation in *K14-Cre;Smad4*^*fl/fl*^ mice. HERS enlarges but cannot elongate to guide root development without Smad4. At the molecular level, Smad4-mediated TGF-β/BMP signaling is required for *Shh* expression in HERS and *Nfic* (nuclear factor Ic) expression in the cranial neural crest (CNC)-derived dental mesenchyme. *Nfic* is crucial for root development, and loss of *Nfic* results in a CNC-derived dentin defect similar to the one of *K14-Cre;Smad4*^*fl/fl*^ mice. Significantly, we show that ectopic Shh induces *Nfic* expression in dental mesenchyme and partially rescues root development in *K14-Cre;Smad4*^*fl/fl*^ mice. Taken together, our study has revealed an important signaling mechanism in which TGF-β/BMP signaling relies on a Smad-dependent mechanism in regulating *Nfic* expression via Shh signaling to control root development. The interaction between HERS and the CNC-derived dental mesenchyme may guide the size, shape, and number of tooth roots. © 2010 American Society for Bone and Mineral Research.

## Introduction

Mammalian ectodermal cells undergo complex reciprocal interactions with underlying mesenchymal cells to form a variety of appendages, including teeth, hair follicles, and mammary glands.(1–3) The tooth organ is an excellent model for studies of heterotypic tissue interactions and the formation of unique dental structures.(1,4–12) Tooth crown formation is controlled by many cytokines and transcription factors that regulate critical gene expression during tooth development.(4,13,14) Compared with the study of crown formation, we have limited information on the molecular regulatory mechanism of root development.

Following crown development, inner enamel epithelium (IEE) and outer enamel epithelium (OEE) form a bilayered epithelial structure termed *Hertwig's epithelial root sheath* (HERS) that migrates apically and participates in root formation and the completion of tooth organ development. Morphologically, HERS is a structural boundary between two dental mesenchymal tissues, the dental papilla and the follicle. Interestingly, HERS breaks up into epithelial rests and cords, allowing dental follicle cells to come in contact with the outer surface of the root during dentin and cementum formation. This strategic position suggests an important role for HERS during critical interactions between dental epithelium and mesenchyme.(15–17)

Previous studies have shown that many genes, including bone morphogenetic proteins (*BMP*s), fibroblast growth factors (*FGF*s), sonic hedgehog (*Shh*), *Notch*, *Gli*, *Msx1, Msx2*, and *Nfic*, are involved in tooth root development.(4,13,14,18,19) *Nfic* (nuclear factor Ic) may have a specific function as a key regulator of root formation because tooth root development is affected most prominently in *Nfic*^*−/−*^ mice.(19,20) However, the mechanism of root formation and the interactions between the epithelial structure (HERS) and mesenchymal tissues (dental papilla and follicle) remain unclear.

The transforming growth factor β (TGF-β) superfamily of cytokines is comprised of TGF-βs, BMPs, activins, and related proteins. TGF-β signaling plays an important role in regulating a broad spectrum of biologic processes such as cell proliferation, differentiation, apoptosis, migration, and extracellular matrix remodeling.(21–23) The canonical TGF-β signaling pathway involves the TGF-β ligand binding to type II and type I receptors. The activated receptor complex phosphorylates Smad proteins (R-Smads), which form a complex with the common Smad (Smad4). The Smad complex then translocates into the nucleus to regulate the expression of an array of target genes.(24) Smad4 is a central mediator of the canonical TGF-β signaling pathway.

To date, few animal models have been identified to explore the function of the HERS directly. In this study we report a rootless animal model, *K14-Cre;Smad4*^*fl/fl*^ mice. Following inactivation of *Smad4* in dental epithelial cells, the development of molar roots is arrested, and the formation of enamel and dentin is also affected severely. Our data suggest that TGF-β/BMP signaling relies on a Smad4-dependent mechanism that regulates *Nfic* expression via Shh signaling. Thus Smad4 controls a genetic hierarchy involving *Shh* and *Nfic* that plays a crucial role in regulating epithelial-mesenchymal interaction during root development.

## Materials and Methods

### Generation of K14*-Cre;Smad4*^*fl/fl*^ and *Nfic*^*−/−*^ mice

Male mice carrying the *K14-Cre* allele(25) were crossed with *Smad4*^*fl/fl*^(26) females to generate *K14-Cre;Smad4*^*fl/+*^ mice. This transgene drives *Cre* expression in basal keratinocytes from E9.5.(27,28,32) The male *K14-Cre;Smad4*^*fl/+*^ mice were mated with *Smad4*^*fl/fl*^ female mice to generate *K14-Cre;Smad4*^*fl/fl*^ null alleles that were genotyped using polymerase chain reaction (PCR) primers as described previously.(33,34) Male and female heterozygous *Nfic*^*+/−*^ mice were crossed to generate *Nfic*^−/−^ progeny, and adult animals were determined by PCR primers as described previously.(19) All samples of *K14-Cre;Smad4*^*fl/fl*^ and *Nfic*^−/−^ mice were fixed in 4% paraformaldehyde and processed into paraffin-embedded serial sections using routine procedures. For general morphology, deparaffinized sections were stained with hematoxylin and eosin (H&E) using standard procedures.

### Kidney capsule transplantation

Kidney capsule transplantation was carried out as described previously.(30,34) The mandibular process of the first branchial arch at E11.5 was dissected from embryos. The explants were cultured for 1 day during genotyping and then grafted under the kidney capsule according to standard procedures. Then 2 and 4 weeks after transplantation, the host mice were killed, and the grafts were processed for histologic analysis. For rescue experiment, Shh and BSA beads were applied to the grafts and transplanted into the capsule with the first branchial arches. Bead preparation was carried out as described previously.(30) Affi-Gel blue agarose beads (Bio-Rad, Hercules, CA, USA) were soaked in recombinant human Shh (100 µg/mL; R&D Systems, Minneapolis, MN, USA) or bovine serum albumin (BSA) (100 µg/mL) overnight at 4°C prior to use.

### In vitro bead implantation

The molar tooth germs from wild-type and *K14-Cre;Smad4*^*fl/fl*^ mandibles at the newborn stage were isolated in cold PBS and placed on filters in Trowell-type organ cultures. Protein-soaked beads were implanted onto the dental mesenchyme in BGJb culture medium (Gibco, Invitrogen, Carlsbad, CA, USA) supplemented with 10% ascorbic acid. Explants were harvested after 2 days in culture, fixed in 4% paraformaldehyde, and processed for whole-mount in situ hybridization and histologic analysis.

### Immunohistochemistry

Tissues were fixed with 4% paraformaldehyde for immunohistochemistry. Paraffin blocks containing processed mouse tissue were sectioned coronally (6 µm in thickness) for immunohistochemical analysis. The slides were heated in a 60°C oven for 30 minutes and subsequently hydrated to water through a series of decreasing concentrations of ethanol. The immunohistochemical staining was performed by using the Zymed HistoStain SP kit (Invitrogen). The specific anti-Smad4 antibody was purchased from Santa Cruz Biotechnology, Inc. (Santa Cruz, CA, USA). It is noted that owing to the background issue, there are some limitations with data interpretation of anti-Smad4 staining. To address this issue, we had performed in situ hybridization analysis to verify the successful tissue-specific inactivation of *Smad4* in neural crest and epithelial cells.(29,31)

### In situ hybridization

Tissues were fixed with 4% paraformaldehyde in PBS, embedded in paraffin, serially sectioned, and mounted following standard procedures. DNA fragments of *Amelogenin*, dentin sialophosphoprotein (*Dspp*), *Nfic*, *Shh*, and *Gli* were subcloned in vector plasmids. Digoxigenin (DIG)–labeled sense and antisense cRNA riboprobes were synthesized using the DIG RNA Labeling Mix (Roche Molecular Biochemicals, Indianapolis, IN, USA). Paraffin-embedded sections were dewaxed and treated with proteinase K (20 µg/mL) and 0.2 M HCl, acetylated, and hybridized overnight with DIG- labeled probes as described previously.(31)

## Results

### Loss of *Smad4* in the dental epithelium affects tooth root formation

**Fig. 1:**
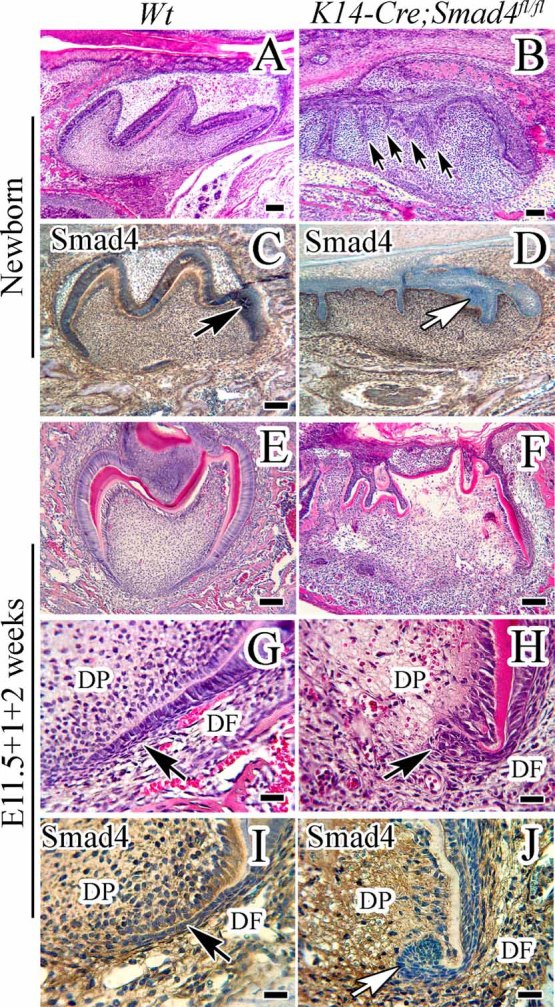
Tooth root development in mice lacking *Smad4*. (*A–D*) H&E staining (*A*, *B*) and immunohistochemistry of Smad4 (*C*, *D*) of sections of teeth from newborn wild-type (Wt) and *K14-Cre;Smad4^fl/fl^* mice. Compared with tooth development in wild type (*A*), *K14-Cre;Smad4^fl/fl^* teeth show more epithelium folding (*B*, *arrows*). *Smad4* is detectable in both dental epithelium and mesenchyme in wild-type mice (*C*, *arrow* indicates the dental epithelium) but undetectable in the dental epithelium of *K14-Cre;Smad4^fl/fl^* mice (*D*, *white arrow*). (*E–J*) H&E staining (*E–H*) and immunohistochemistry of Smad4 (*I*, *J*) of E11.5 tooth germs cultured in kidney capsules. After 2 weeks of cultivation under the kidney capsule, the beginning of root formation and well-organized enamel and dentin are detectable in wild-type samples. Note that the HERS is elongated vertically (*G*, *arrow*). In *K14-Cre;Smad4^fl/fl^* mice, HERS is enlarged and does not grow out of the crown (*H*, *J*, *arrows*). Smad4, which is detectable in wild-type mice (*I*, arrow indicates the dental epithelium), is undetectable in the dental epithelium of *K14-Cre;Smad4^fl/fl^* mice (*J*, *white arrow*). Well-organized enamel and dentin are seldom found in *K14-Cre;Smad4^fl/fl^* mice (*F*). DF = dental follicle; DP = dental pulp. Scale bars (*A–F*) = 100 µm; scale bars (*G–J*) = 25 µm.

**Fig. 2:**
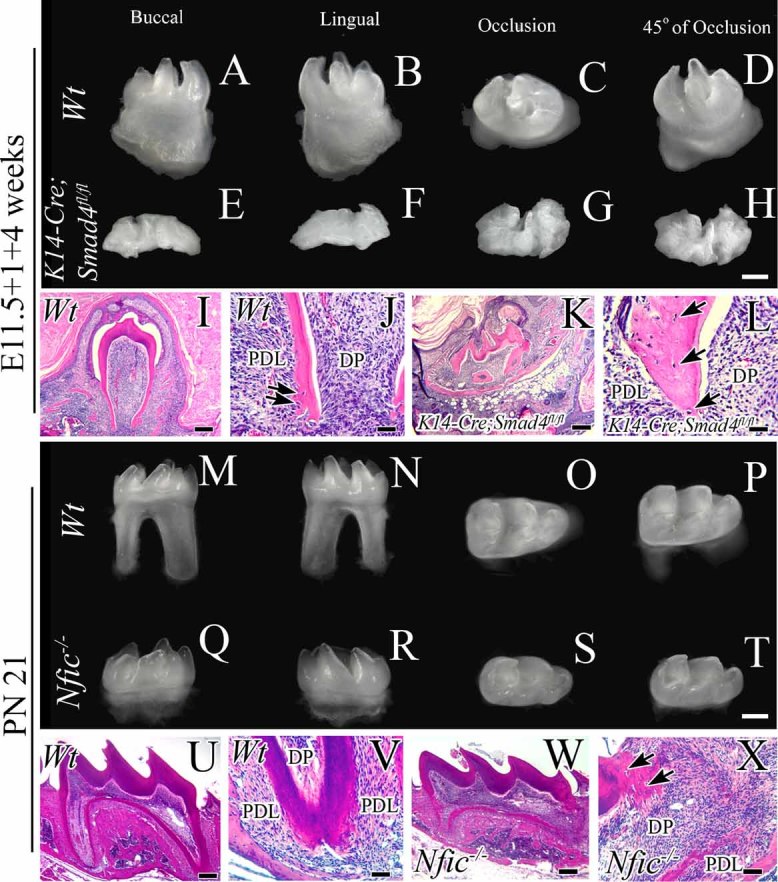
Root development in *K14-Cre;Smad4^fl/fl^* and *Nfic^−/−^* mice. (*A–L*) Whole-mount views and sections stained with H&E of tooth germs from wild-type (Wt) and *K14-Cre;Smad4^fl/fl^* mice cultured for 4 weeks in kidney capsules. After 4 weeks of cultivation in the kidney capsule, the mineralized teeth of wild-type mice are well developed, with normal cusps and root formation (*A–D*). Root dentin and cementum are also identifiable with H&E staining (*I*, *J*, *arrows* indicate the cementocytes). Root formation is not detectable in *K14-Cre;Smad4^fl/fl^* mice (*E–H*), and odontoblasts are embedded in the dentin to form cellular dentin (*K*, *L*, *arrows* indicate the cells in dentin). (M–X) Whole-mount views and sections stained with H&E of tooth germs from postnatal day 21 wild-type and *Nfic*^−/−^ mice. Two roots are clearly identifiable in wild-type teeth (*M–P*, *U*, *V*). *Nfic*^−/−^ mice do not develop a normal tooth root, and cellular dentin is detectable in root-like areas by H&E staining (*Q–Y*, *W*, *X*, *arrows* indicate the cells in dentin). The crown and cusps are normal in *Nfic*^−/−^ teeth. DP = dental pulp; PDL = periodontal ligament. Scale bars (*A–I*, *K*, *M–U*, *W*) = 100 µm; scale bars (*J*, *L*, *V*, *X*) = 25 µm.

We had previously generated mice with a conditional *Smad4* knockout in the dental epithelial cells (*K14-Cre;Smad4*^*fl/fl*^)(23) to explore the functional significance of *Smad4* in regulating the fate of the dental epithelium and tooth development. Unfortunately, *K14-Cre;Smad4*^*fl/fl*^ mice die at birth, so we cannot investigate the role of *Smad4* in regulating root development at postnatal stages. Therefore, we transplanted the mandible containing the lower first molars from E11.5 *K14-Cre;Smad4*^*fl/fl*^ mice into a kidney capsule to observe root formation. At the newborn stage, the morphology of teeth in *K14-Cre;Smad4*^*fl/fl*^ mice was affected (Fig. 1*A, B*), as described previously.(31) To confirm the loss of *Smad4* in *K14-Cre;Smad4*^*fl/fl*^ mice, we assayed the expression of *Smad4* using immunohistochemistry. In wild-type teeth, *Smad4* is strongly expressed in the dental epithelium and mesenchyme at the newborn stage and after 2 weeks in the kidney capsule (Fig. 1*C, I*) but is undetectable in the dental epithelium of *K14-Cre;Smad4*^*fl/fl*^ mice (Fig. 1*D, J*). These data confirm that *Smad4* is specifically inactivated in the dental epithelium of *K14-Cre;Smad4*^*fl/fl*^ mice. After 2 weeks of cultivation in the kidney capsule, the mineralized teeth of the wild-type sample were well developed, with normal cusps and initiation of root formation (Fig. 1*E, G*). The HERS is formed by two layers of dental epithelial cells located in the apical part of the tooth (Fig. 1*G*, *arrow*). However, in *K14-Cre;Smad4*^*fl/fl*^ samples, we did not observe root formation (Fig. 1*F, H*). Morphogenesis of the first mandible molar was severely disrupted. The HERS was enlarged, and HERS cells failed to grow out of the crown in *K14-Cre;Smad4*^*fl/fl*^ samples (Fig. 1*H, J*, *arrows*). After 4 weeks of cultivation in the kidney capsule, wild-type teeth and roots developed well (Fig. 2*A–D*). We could clearly detect root dentin and cementum with H&E staining (Fig. 2*I, J*, cementocytes are indicated by *arrows*). In contrast, we still did not observe root formation in *K14-Cre;Smad4*^*fl/fl*^ samples (Fig. 2*E–H, K, L*). The first mandible molar appeared translucent, and the enamel was not well-organized. Dentin formation appeared abnormal. Odontoblasts were embedded into the dentin, an abnormal condition not observed in wild-type samples (Fig. 2*L*, *arrows*).

### Abnormal enamel and dentin formation in *K14-Cre;Smad4*^*fl/fl*^ mice

**Fig. 3:**
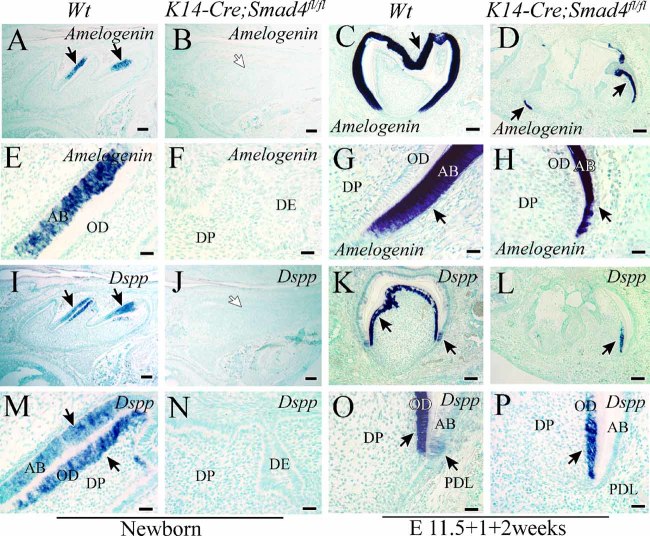
Expression of *Amelogenin* and *Dspp* is delayed in *K14-Cre;Smad4^fl/fl^* teeth. In situ hybridization of *Amelogenin* (*A–H*) and *Dspp* (*I–P*) in wild-type (Wt) and *K14-Cre;Smad4^fl/fl^* teeth at the newborn stage (*A*, *B*, *E*, *F*, *I*, *J*, *M*, *N*) and 2 weeks after culture in kidney capsules (*C*, *D*, *G*, *H*, *K*, *L*, *O*, *P*). In wild-type mice, *Amelogenin* expression was detectable in all ameloblasts, and *Dspp* was detectable in the odontoblast layer and in some ameloblasts (*A*, *B*, *E*, *F*, *I*, *J*, *M*, *N*, *arrows*). In *K14-Cre;Smad4^fl/fl^* mice, gene expression of *Amelogenin* and *Dspp* could not be detected at the newborn stage (*B*, *F*, *J*, *N*, *white arrows*) and was dramatically reduced after 2 weeks in the kidney capsule (*D*, *H*, *L*, *P*, *arrows*). AB = ameloblast; DE = dental epithelium; DP = dental pulp; HERS = Hertwig's epithelial root sheath; OD = odontoblast. Scale bars (*A–D*, *I–L*) = 100 µm; scale bars (*E–H, M–P*) = 25 µm.

The enamel and dentin did not develop well in *K14-Cre;Smad4*^*fl/fl*^ mice. After 2 weeks of cultivation under the kidney capsule, we observed well-organized enamel and dentin in wild-type samples (Fig. 1*E*) but not in *K14-Cre;Smad4*^*fl/fl*^ samples (Fig. 1*F*). *Amelogenin* and *Dspp* have been used extensively as differentiation markers for ameloblasts and odontoblasts, respectively.(30) To examine the status of ameloblast and odontoblast differentiation in *K14-Cre;Smad4*^*fl/fl*^ mice, we assayed the expression of *Amelogenin* and *Dspp* using in situ hybridization. In wild-type mice, we detected *Amelogenin* expression in all ameloblasts, and *Dspp* was present in the odontoblast layer and in some ameloblasts at the newborn stage in vivo (Fig. 3*A, E, I, M*) and on day 14 after kidney capsule transplantation (Fig. 3*C, G, K, O*). In *K14-Cre;Smad4*^*fl/fl*^ mice, however, gene expression of *Amelogenin* and *Dspp* was not detectable at the newborn stage (Fig. 3*B, F, J, N*) and was dramatically reduced on day 14 after kidney capsule transplantation (Fig. 3*D, H, L, P*). Our data suggest that *Smad4* is important for both enamel and dentin formation in the dental epithelium during tooth development but is not required for ameloblast and odontoblast differentiation.

### *Smad4* is required in the dental epithelium for *Nfic* expression in the dental mesenchyme during root development

**Fig. 4:**
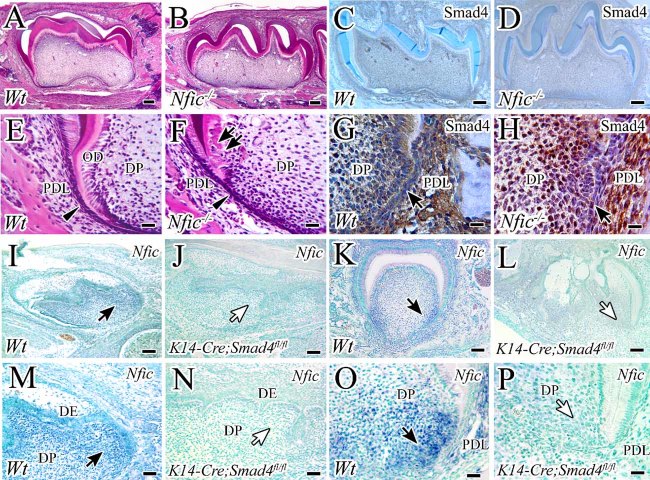
Expression of *Nfic* is affected in *K14-Cre;Smad4^fl/fl^* teeth. (*A–H*) H&E staining (*A*, *B*, *E*, *F*) and immunohistochemistry of *Smad4* (*C*, *D*, *G*, *H*) in postnatal day 7 wild-type (Wt) and *Nfic*^−/−^ mice. HERS is detectable in wild-type and *Nfic*^−/−^ mice (*A*, *B*, *E*, *F*, *arrow heads*). The crown and cusp appear unaffected, but the root does not develop well in *Nfic*^−/−^ mice, and cellular dentin is detectable (*B*, *F*, *arrows*). *Smad4* expression is indistinguishable in wild-type and *Nfic*^−/−^ mice (*C*, *D*, *G*, *H*, *arrows*). (*I–P*) In situ hybridization of *Nfic* in wild-type and *K14-Cre;Smad4^fl/fl^* newborn teeth (*I*, *J*, *M*, *N*) and tooth germs cultured for 2 weeks in kidney capsules (*K*, *L*, *O*, *P*). *Nfic* expression is detectable in preodontoblasts, odontoblasts, and dental pulp in wild-type mice (*I*, *M*, *K*, *O*, *black arrows*) but undetectable in *K14-Cre;Smad4^fl/fl^* mice (*J*, *L*, *N*, *P*, *white arrows*). DP = dental pulp; PDL = periodontal ligament. Scale bars (*A–D*, *I–L*) = 100 µm; scale bars (*M*, *N*) = 50 µm; scale bars (*E–H*, *O*, *P*) = 25 µm.

To investigate the signaling mechanisms of *Smad4* in regulating epithelial–mesenchymal interactions during root development, we surveyed expression patterns of genes that are critical during early root formation. Because we observed the beginning of root formation after 2 weeks of cultivation under the kidney capsule (Fig. 1*E, G*), we compared gene expression patterns in *K14-Cre;Smad4*^*fl/fl*^ and wild-type mice during that time period. The nuclear factor I (Nfi) family of transcription-replication factors is encoded by four genes in mammals and plays an important role in regulating the expression of many cellular genes. The four *Nfi* genes have unique functions and do not function redundantly.(32,33) Loss of *Nfic* results in major defects in postnatal murine tooth development and absence of molar root formation.(19) We confirmed that *Nfic*^−/−^ mice fail to develop a normal tooth root (Fig. 2*Q–T*), which is present in wild-type mice on postnatal day 21, (Fig. 2*M–P*). We next examined root dentin formation and found abnormal cellular dentin in rootlike areas in *Nfic*^*−/−*^ mice on postnatal day 7, although the dental crown and cusp are normal (Fig. 4*A, B, E, F*). *Nfic* is required for root development, especially root dentin formation, but not for crown formation. Furthermore, we found that defects of crown and root development in *K14-Cre;Smad4*^*fl/fl*^ mice are more severe than those of *Nfic*^*−/−*^ mice (Fig. 2*E–H, Q–T*). Therefore, we hypothesized that expression of *Nfic* in the dental mesenchyme may require *Smad4* in the dental epithelium. The expression of *Nfic*, which was clearly detectable in preodontoblasts, odontoblasts, and dental pulp in wild-type mice (Fig. 4*I, K, M, O*), was not detectable in *K14-Cre;Smad4*^*fl/fl*^ mice at the newborn stage and on day 14 after kidney capsule transplantation (Fig. 4*J, L, N, P*). In contrast, *Smad4* expression could be identified in both wild-type and *Nfic*^*−/−*^ mice (Fig. 4*C, D, G, H*). Our data suggest that *Smad4* is required in the dental epithelium for *Nfic* expression in the dental mesenchyme.

### *Smad4* is required for *Shh* expression during root development

**Fig. 5:**
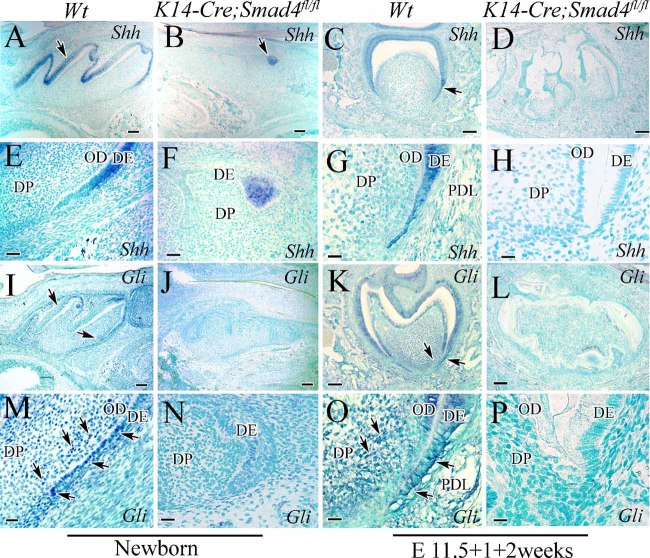
*Shh* and *Gli* expression in wild-type and *K14-Cre;Smad4^fl/fl^* mice. In situ hybridization of *Shh* (*A–H*) and *Gli* (*I–P*) in newborn teeth (*A*, *B*, *E*, *F*, *I*, *J*, *M*, *N*) and tooth germs cultured 2 weeks in kidney capsules (*C*, *D*, *G*, *H*, *K*, *L*, *O*, *P*) from wild-type (Wt) and *K14-Cre;Smad4^fl/fl^* mice. In wild-type mice, *Shh* is expressed in dental epithelium at the newborn stage and in ameloblasts and HERS cells after 2 weeks of kidney capsule culture (*A*, *E*, *C*, *G*, *arrows*). In *K14-Cre;Smad4^fl/fl^* newborn mice, *Shh* expression is reduced dramatically (*B*, *F*) and is undetectable in dental epithelial cells, including HERS, after 2 weeks of kidney capsule culture (*D*, *H*). *Gli* is expressed in the dental epithelium and dental mesenchyme in wild-type mice (*I*, *M*, *K*, *O*, *arrows*) but is undetectable in the *K14-Cre;Smad4^fl/fl^* mice (*J*, *N*, *L*, *P*). AB = ameloblast; DE = dental epithelium; DP = dental pulp; OD = odontoblast, PDL = periodontal ligament. Scale bars (*A–D*, *I–L*) = 100 µm; scale bars (*E–H*, *M–P*) = 25 µm.

*Smad4* is an intracellular transcription factor, and its activity in the dental epithelium cannot directly affect *Nfic* expression in the dental mesenchyme. Therefore, we infer that some *Smad4*-dependent factor(s) must be released from the dental epithelium to control *Nfic* expression in the dental mesenchyme. *Shh*, a member of the hedgehog signaling family, is expressed in the dental epithelium and plays an essential role during tooth development.(34,35) Previous studies have suggested that *Shh* is induced by BMP signaling during tooth development.(36) We hypothesized that *Shh* might be one of the key factors controlled by *Smad4* in the dental epithelium to affect gene expression in the dental mesenchyme. We found that *Shh* is expressed in ameloblasts and HERS cells in wild-type mice (Fig. 5*A, C, E, G*). *Gli1*, a transcription factor activated by *Shh*,(37) is also expressed in the dental epithelium and dental mesenchyme at the newborn stage and after 2 weeks of culture in a kidney capsule (Fig. 5*I, K, M, O*). In contrast, we could not detect *Shh* signaling in dental epithelial cells including HERS from *K14-Cre;Smad4*^*fl/fl*^ mice after culture in a kidney capsule for 2 weeks (Fig. 5*D, H*). We found that *Shh* expression was dramatically reduced (Fig. 5*B, F*) and failed to detect *Gli1* expression in newborn *K14-Cre;Smad4*^*fl/f*^ mice (Fig. 5*J, L, N, P*). Our data suggest that *Shh* is a downstream target regulated by TGF-β/BMP signaling during root development.

### Partial rescue of root development in *K14-Cre;Smad4*^*fl/fl*^ tooth germs by ectopic Shh protein

**Fig. 6:**
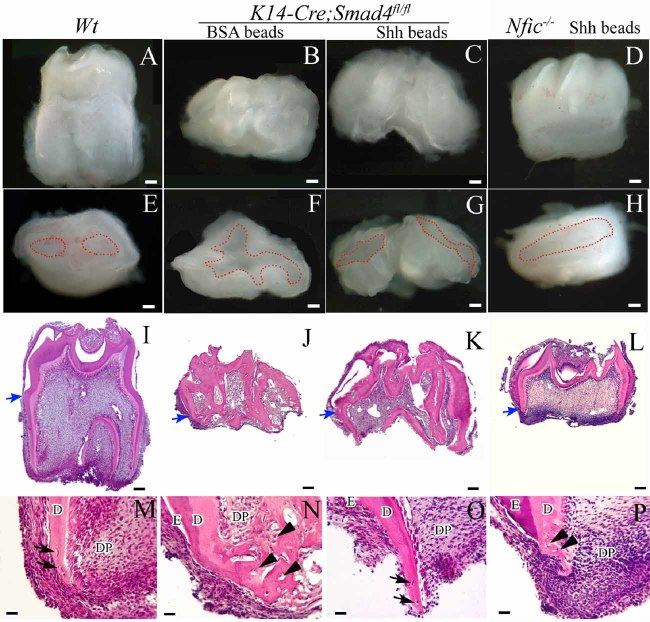
Ectopic Shh partially rescues the root defect in *K14-Cre;Smad4^fl/fl^* mice. Whole-mount views (*A–H*) and sections stained with H&E (*I–P*) of tooth germs from wild-type (Wt), *K14-Cre;Smad4^fl/fl^*, and *Nfic*^−/−^ mice cultured for 4 weeks in kidney capsules with BSA or Shh beads. Red-dotted sections in panels *E–H* outline the roots. In wild-type samples, the root developed well; two roots, dentin, cementum, and the CEJ are clearly identifiable (*A*, *E*, *I*, *M*; *blue arrow* indicates the CEJ; *black arrows* indicate cementocytes). In *K14-Cre;Smad4^fl/fl^* samples treated with BSA beads, the root is undetectable, and abnormal cellular dentin is detectable (*B*, *F*, *J*, *L*; *blue arrow* indicates the CEJ; *black arrowheads* indicate the cellular dentin). In *K14-Cre;Smad4^fl/fl^* samples treated with Shh beads, root formation is detectable, but shorter than that in wild-type samples (*C*, *G*, *K*, *O*). In addition, root dentin is normal, cementum is detectable on the surface of root (*O*, *black arrows* indicate cementocytes), and the CEJ is clearly identifiable (*K*, *blue arrow* indicates the CEJ). In *Nfic^−/−^* samples treated with Shh beads, the root is hard to identify; abnormal cellular dentin is detectable (*D*, *H*, *L*, *P*, *blue arrow* indicates the CEJ; *black arrowheads* indicate the cellular dentin). E = enamel; D = dentin; DP = dental pulp. Scale bars (*A–L*) = 100 µm; scale bars (*M–P*) = 25 µm.

Because *Shh* is downregulated in the absence of *Smad4*, we tried to use ectopic Shh to rescue the root defect in *K14-Cre;Smad4*^*fl/fl*^ tooth organs. After we added Shh beads into the kidney capsule for 4 weeks, we observed root formation in *K14-Cre;Smad4*^*fl/fl*^ samples. Although the roots were not as long as in wild-type mice (Fig. 6*A, E*), we detected the development of two roots in *K14-Cre;Smad4*^*fl/fl*^ samples with Shh beads (Fig. 6*C, G*). The cementum-enamel junction (CEJ), the boundary between the crown and the root, can be visualized with H&E staining after kidney capsule culture of wild-type samples (Fig. 6*I–L*, *blue arrows*). The CEJ and root structure are clearly identifiable in *K14-Cre;Smad4*^*fl/fl*^ samples treated with Shh beads (Fig. 6*G, K*). Moreover, we detected normal dentin, but not cellular dentin, and cementum on the surface of the rescued root after addition of ectopic Shh (Fig. 6*K, O*, *arrows* indicate the cementocytes), as we did in wild-type samples (Fig. 6*I, M*, *arrows* indicate the cementocytes). We could not find root structures in *K14-Cre;Smad4*^*fl/fl*^ samples treated with BSA beads (Fig. 6*B, F, J, N*, *arrowheads* indicate the cellular dentin). Shh could not rescue the root defect in *Nfic*^*−/−*^ samples (Fig. 6*D, H, L, P*, *arrowheads* indicate the cellular dentin), suggesting that *Nfic* acts downstream of *Shh* signaling to control root formation. Our rescue experiments strongly suggest that *Shh* is a downstream target of TGF-β/BMP signaling that controls root development.

### Shh induces *Nfic* expression during root formation

**Fig. 7:**
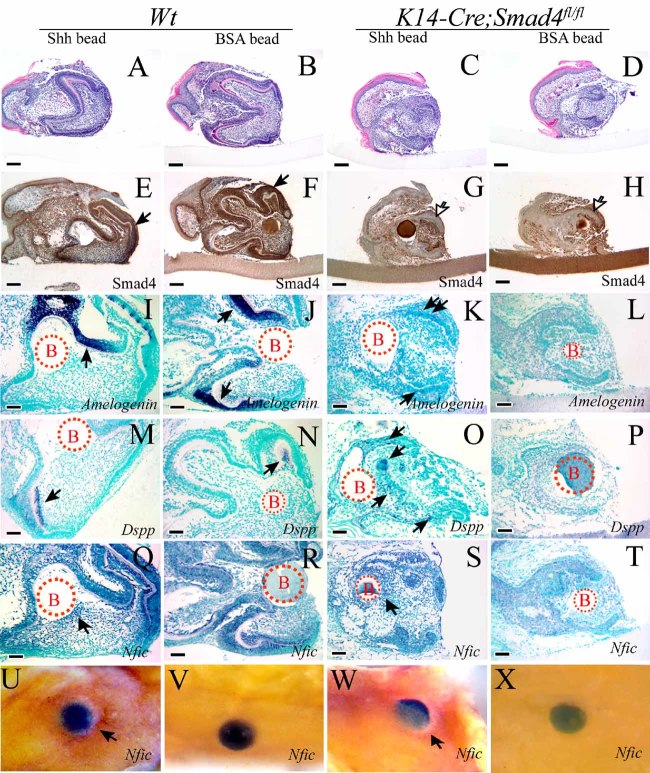
Rescue of *Nfic* gene expression by ectopic Shh in *K14-Cre;Smad4^fl/fl^* mice. Analysis of gene expression in newborn wild-type (Wt) and *K14-Cre;Smad4^fl/fl^* tooth germs cultured with Shh and BSA beads in dental pulp for 2 days. (*A–D*) H&E staining. (*E–H*) Immunohistochemistry with *Smad4* antibody. *Smad4* expression is not detectable in the dental epithelium of *K14-Cre;Smad4^fl/fl^* tooth germs. (*I–X*) In situ hybridization of *Amelogenin* (*I–L*), *Dspp* (*M-P*), and *Nfic* (*Q–X*) of sections (*I-T*) and in whole mount (*Nfic* expression, *U–X*). Expression of *Amelogenin* and *Dspp* is clearly detectable in wild-type samples (*I*, *J*, *M*, *N*). Expression of *Amelogenin* and *Dspp* is not detectable in tooth germ of *K14-Cre;Smad4^fl/fl^* mice treated with BSA beads (*L*, *P*) but is modestly detectable following treatment with Shh beads (*K, O*, *arrows* indicate positive signal). *Nfic* expression is detectable around the Shh beads in the dental pulp of both wild-type and *K14-Cre;Smad4^fl/fl^* mice (*Q*, *S*, *U*, *W*, arrows indicate positive signal), whereas *Nfic* expression is undetectable in *K14-Cre;Smad4^fl/fl^* samples treated with BSA beads (*T*, *X*). Scale bars (*A–H*) = 100 µm; scale bars (*I–T*) = 50 µm.

In order to explore the mechanism of Shh rescue of the root defect, we cultured newborn *K14-Cre;Smad4*^*fl/fl*^ tooth germ with Shh beads in dental pulp. We used H&E staining (Fig. 7*A–D*) and immunohistochemistry of Smad4 (Fig. 7*E–H*) to confirm that the dental epithelium of the mutant samples was Smad4-negative. We detected *Nfic* expression around the Shh beads in the dental pulp of both wild-type and *K14-Cre;Smad4*^*fl/fl*^ tooth germs by whole-mount and section in situ hybridization (Fig. 7*Q, S, U, W*, *arrows*), whereas we failed to detect *Nfic* expression in *K14-Cre;Smad4*^*fl/fl*^ samples with BSA beads (Fig. 7*T, X*). Moreover, *Amelogenin* and *Dspp* are also expressed in *K14-Cre;Smad4*^*fl/fl*^ samples treated with Shh beads (Fig. 7*I, K, M, O*) but undetectable with BSA beads (Fig. 7*J, L, N, P*). Our data suggest that Shh induces *Nfic*, *Amelogenin*, and *Dspp* expression during tooth and root formation.

## Discussion

Organogenesis is the process by which the ectoderm, endoderm, and mesoderm interact with each other and develop into a complex functional unit. An understanding of organogenesis and its regulation is of paramount importance in developmental biology. Tooth organ development is a good model for studies of heterotypic tissue interactions, including crown and root formation. Tooth crown formation is a multistep process that is mediated through a series of epithelial-mesenchymal interactions.(1,22) Although BMP, Shh, Wnt, and FGF are expressed in the developing root, the molecular regulatory mechanism of root development remains unknown. During root development, the transition from dental cervical loop to HERS is generally regarded as the beginning of root formation.(38,39) However, the functional mechanism of HERS in guiding root development is still unclear.(38–41) In this study we investigated the functional significance of TGF-β/BMP signaling in regulating the fate of HERS cells and epithelial-mesenchymal interaction during root development.

TGF-β/BMP signaling has been shown to function in the initiation of tooth and root development.(4,13,14,18) *Bmp2*, *-3*, *-4*, and -*7* are detected during root development, and inhibition of BMP signaling in *K14-Noggin* mice results in a severe root-development defect.(42,43) Smad4, the central mediator for the canonical TGF-β/BMP signaling pathway, plays a crucial role in regulating tooth development.(31) In order to investigate the functional significance of TGF-β/BMP signaling in dental epithelial cells during root development, we used *K14-Cre;Smad4*^*fl/fl*^ mice specifically lacking *Smad4* expression in dental epithelial cells. We found that root formation is arrested at the initiation stage in *K14-Cre;Smad4*^*fl/fl*^ mice. The HERS fails to form its normal continuous bilayer structure and to grow apically. Instead, the cervical loop of the dental epithelium is enlarged in *K14-Cre;Smad4*^*fl/fl*^ mice. TGF-β and BMP ligands are expressed in both dental epithelium and mesenchyme,(30,42,44) but without *Smad4* in the dental epithelium, TGF-β/BMP signaling fails to induce root formation. Therefore, we conclude that the elongated HERS plays an essential role during root development and that *Smad4* in dental epithelial cells is required for tooth root formation.

Morphogenesis of the tooth crown is governed by interactions between the oral ectoderm and neural crest–derived ectomesenchyme.(1,10,11,12,45) During root development, continuous HERS cells are located between the dental papilla and follicle, which are both derived from cranial neural crest. The special sandwich-like structure indicates a possible biologic significance of interaction between the dental epithelium and mesenchyme. In fact, HERS may function as an essential structure for the guidance of root formation.

In this study we compared two rootless animal models, *Nfic*^−/−^ mice (*Nfic* is expressed primarily in dental mesenchyme) and *K14-Cre;Smad4*^*fl/fl*^ mice, to investigate the tissue-tissue interaction and signaling-pathway cascade during root formation. We found that *Smad4* is required in the HERS for *Nfic* expression in the dental mesenchyme. Therefore, we propose that interaction between HERS and dental mesenchymal cells controlled by TGF-β/BMP signaling is essential for root dentin formation.

**Fig. 8:**
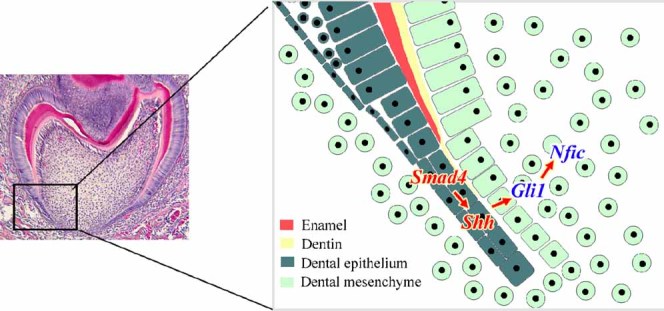
A Smad4-Shh-Gli1-Nfic signaling pathway mediates molar root development. Schematic diagram of our model for the Smad4 signaling pathway during tooth root development. *Smad4* in the dental epithelium is required for *Shh* expression. Then Shh is released from the dental epithelium, acts through Gli1, and induces *Nfic* expression in the dental mesenchyme for normal root formation.

We also found that the expression of *Shh* and its transcription factor, *Gli1*, is reduced dramatically in *K14-Cre;Smad4*^*fl/fl*^ mice. Shh signaling is important for tooth development.(34,35,46–49) Previous studies also reported that Bmp signaling induces *Shh* expression(36) and that *Smad4* is required for *Shh* expression in dental cusp epithelium during crown formation.(31) Loss of *Ptc1*, the receptor for Shh, results in a short molar root, which indicates the significance of *Shh* signaling during root development.(49) In this study we investigate the functional significance of Shh signaling in mediating epithelial and mesenchymal interaction during root development. After culturing tooth germs with ectopic Shh in a kidney capsule, we found that not only is the root defect in *K14-Cre;Smad4*^*fl/fl*^ mice partially rescued but also *Nfic* expression and abnormal cellular dentin are restored to normal. However, ectopic Shh is unable to rescue the root defect in *Nfic*^*−/−*^ mice. Taken together, we show that *Smad4* in the dental epithelium is required for *Shh* expression. Shh released from the dental epithelium acts through *Gli1* in the dental mesenchyme to induce *Nfic* expression to control root development (Fig. 8). Our model clearly demonstrates that the Smad4-Shh-Nfic signaling network plays a crucial role in mediating epithelial-mesenchymal interaction to control root development.

As an ectodermal appendage, tooth organ development involves crown and root formation. Crown pattern formation is almost completed by birth. Tooth root development begins following crown formation and lasts almost 3 weeks after birth in mice. To date, the transition from crown to root formation during development has remained undefined. Previous studies have shown that the regulatory mechanisms of crown and root development are not identical.(19,50) For example, *Nfic* is specifically required for root dentin development and root formation but is not involved in crown formation.(19) Intriguingly, however, HERS formation is not affected in *Nfic*^*−/−*^ mice. Previous study shows that the disappearance of Fgf10 signaling leads to the transition from crown to root formation owing to a loss of the dental epithelial stem cell compartment.(51) Overexpression of Fgf10 results in enlargement of the HERS and failure to form root. We also observed enlarged HERS in *K14-Cre;Smad4*^*fl/fl*^ mice, suggesting that TGF-β/BMP signaling in the dental epithelium is involved in HERS elongation. *Smad4*-mediated TGF-β/BMP signaling in the dental epithelium is required for *Nfic* expression in the dental mesenchyme. In parallel, our data show that TGF-β/BMP signaling in dental epithelial cells inhibits Fgf10 signaling in the dental mesenchyme (data not shown), suggesting that TGF-β/BMP signaling in the dental epithelium may play a key role in the initiation stage of root formation. *Smad4* is the central mediator of the canonical TGF-β signaling pathway. TGF-β2, BMP-2, -4, -5, and -7, and activin are expressed in dental epithelium and/or mesenchyme during tooth and root development. The functional significance of these ligands in regulating root development awaits further investigation.

In summary, our study suggests that the elongation of dental epithelial cells, HERS, is essential for root development and that TGF-β/BMP signaling relies on a *Smad4*-dependent signaling mechanism to regulate *Nfic* expression via Shh signaling to control root formation. HERS cells may provide instructive signaling to control the size, shape, and number of roots. Root development involved dentin and cementum formation and serves as an ideal model for the investigation of epithelial-mesenchymal interaction in regulating postnatal organogenesis. From a clinical perspective, normal root development is critical for the function of dentition. A clear understanding of the regulatory mechanism of root development will provide a comprehensive knowledge of tooth morphogenesis. Furthermore, information generated from this study will lay the foundation for root regeneration, which can be used to support prosthetic restorations to restore dentition in order to provide a biologic solution for a biologic problem.
